# Strangers in a strange land: how diversity professionals navigate their marginal leadership identity

**DOI:** 10.3389/fpsyg.2024.1484472

**Published:** 2024-12-10

**Authors:** Bruno Felix, Mariana Clementino Brandão, Jasmin Mahadevan, Anja Schmitz, Samir Lótfi Vaz, Hélio Arthur Reis Irigaray

**Affiliations:** ^1^Fucape Business School, Espírito Santo, Brazil; ^2^Pforzheim University, Pforzheim, Germany; ^3^Fundação Dom Cabral, Nova Lima, Minas Gerais, Brazil; ^4^Fundação Getúlio Vargas, Rio de Janeiro, Rio de Janeiro, Brazil

**Keywords:** marginal leadership, identity threats, diversity and inclusion, identities, boundary theory, paradoxes

## Abstract

The purpose of the present study was to understand how executives responsible for Diversity and Inclusion construct their identities while occupying positions of “Marginal Leadership,” and how they deal with threats to such identities. We conducted qualitative and inductive research with Brazilian executives in 66 organizations, focusing on their experiences as leaders. In our resulting model, we theorized that leaders in positions of Marginal Leadership demonstrate little role clarity, resources, and confidence for the exercise of their activities, suffering threats to their identities, which are not observed in executives occupying positions in more traditional functions such as Finance, Production, and Marketing. Furthermore, we identified that Brazilian executives responsible for Diversity and Inclusion build three types of Relational Leadership in interaction with individuals in historically disadvantaged positions, and alongside executives in traditional positions. First, “Business Partner,” focusing on performance—boundary segmentation. Second, “Injustice Repairer,” focusing on inclusion—boundary segmentation. Third, “Paradox Manager,” focusing on performance and inclusion—boundary integration. Finally, we found that threats to their identities vary according to the type of Marginal Leadership constructed. While “Business Partners” and “Injustice Repairers” tend to be seen as false representatives or politically inept, the “Paradox Manager” tends to be labeled as a “Fence Sitter.” This study advances the understanding of role identities in positions with ambiguous expectations, integrating Identity Theory and Boundary Theory to explore how diversity leaders integrate their identities in the light of conflicting demands.

## 1 Introduction

Diversity management has become an integral leadership task in many contemporary organizations (Primecz and Mahadevan, 2024). However, what is not yet clearly developed is an understanding of how those who are responsible for matters of diversity, equity/equality and inclusion (EDI) in their organizations (diversity leaders), navigate their identity as “diversity leaders,” also in relation to those “classic” and potentially more mainstream leaders whom they seek to influence for achieving their organization’s EDI goals. We assume that this is not an easy identity to navigate, due to the following reasons: First of all, the goals associated with managing DEI are conflicting in themselves (Spaaij et al., 2020): On the one hand, diversity leaders should shape a ‘fairer’ corporate environment, advance corporate social responsibility and include previously neglected groups (Nkomo et al., 2019). At the same time, they need to fulfill the promises of the so called “business case for diversity,” namely to make organizations more profitable, innovative, agile or customer-oriented by achieving equity and inclusion for more groups (Primecz and Mahadevan, 2024). Secondly, organizations often promote a general discourse that promotes respect for diversity, learning, ethics, and sustainability (Petriglieri and Peshkam, 2022) as key leadership qualities. Thus, high hopes in the area of social sustainability are placed on diversity leaders, yet, in practice, the organization might not provide the conditions required for putting these ideals into practice (Carrillo Arciniega, 2021). This suggests that organizations might not place enough importance on DEI compared to other leadership functions and fields, which results in unfulfilled DEI promises in practice (Carrillo Arciniega, 2021). Consequently, diversity leaders often find themselves at the fringes of organizational leadership (Dennissen et al., 2019; Janssens and Steyaert, 2019); they are supported in theory, but seldom in practice (Martins, 2020); and they operate from a weaker organizational power-base, compared to other leadership functions (Greene and Kirton, 2023). In other words, they are organizationally marginal leaders (Fitzsimmons et al., 2013; based on Park, 1928, 1937). Marginal leaders are those who find themselves between two identities or value-sets and need to devise strategies for, on the one hand, retaining the unique knowledge base which their marginality provides them with, and for, on the other hand, for being sufficiently included in majority practice and decision-making to have an impact onto the organization. Against this background, we ask the question: “How do marginal diversity leaders in organizations respond to paradoxical pressures and doubts about their professional goals?”

We conducted a qualitative and inductive study with Brazilian executives responsible for Diversity and Inclusion in 66 organizations to provide insights for closing this research gap, focusing on their experience as leaders. The study aimed to understand the different Marginal Leadership identities experienced by such executives, as well as threats to these identities. Although they occupied formal leadership positions at higher hierarchical levels, the interviewees characterized themselves as marginal leaders—insiders with weak ties to the establishment, caught between conflicting goals and values (Petriglieri and Peshkam, 2022). The lack of clear boundaries left these leaders exposed to contrasting expectations in the exercise of their roles. In navigating the context of these paradoxes (Noon and Ogbonna, 2021), some participants reported what we interpret as the construction of a paradoxical occupational identity, while others oscillated between humanistic and pragmatic identities. In our model, we also identified the most recurrent threats to identity in each of these cases.

The contribution of our paper lies in introducing the highly relevant concept of marginality to the question of why and how DEI initiatives fail or succeed in practice. Whereas marginality has been applied to leadership in the field of global and international leadership, it has not yet been applied to understand how the marginality of certain leadership positions and functions, such as DEI, shapes overall organizational leadership realities. As a major finding, we suggest that it is the different ways in which diversity leaders navigate their marginal identities that shapes the concrete outcome of diversity goals and policies in organizations.

In order to make this contribution, we proceed as follows: First, we review the relevant literature and provide an overview on methods of data collection and analysis. Next, we present and discuss our findings and derive implications for theory and practice from there. Finally, we summarize and conclude.

Despite the importance of this topic, there is a need to address the following research question:

## 2 Literature review

### 2.1 Leader identity and identity threat

The construction of leadership identity involves multiple levels of self-construction, including individual, relational, and collective (Brewer and Gardner, 1996). The approach adopted in this study proposes a conception that encompasses these three levels, recognizing that leadership is a social and mutually influential process (Petriglieri and Peshkam, 2022). The individual internalization of leader or follower identity is the first element of this process, in which individuals incorporate the identity of leader or follower into their self-concept (DeRue et al., 2009). Next, relational recognition comes into play, highlighting the importance of the perceived relationship between individuals (Sluss and Ashforth, 2007). Additionally, collective endorsement of leadership identity further reinforces this construction by situating the individual within a socially recognized group as leaders or followers (Brewer and Gardner, 1996).

Considering leadership identity in these three dimensions, we recognize that leadership development is linked to relationship building. This contrasts with a static and hierarchical conception of leadership, emphasizing the social and relational dynamics involved in forming leadership identity (Petriglieri and Peshkam, 2022). This approach suggests that leadership is a reciprocal relationship, emphasizing the importance of social interactions in the joint construction of leader and follower identities. In doing so, we engage in an existing discussion on how it happens that one is socially accepted and self-identifies as leader, thus enriching the understanding of the fluid and social nature of leadership (Collinson, 2005).

To meet recent calls to define leadership as a process of social and mutual influence, rather than focused on a leader-centric view or role-based approach (see Uhl-Bien et al., 2014), it is crucial for leadership theories to clarify the mechanisms by which relationships and leadership identities are formed. In this article, we draw on previous research that theorizes a process by which individuals collaboratively develop leadership relationships, explaining why some are perceived as leaders while others as followers (DeRue and Ashford, 2010; Epitropaki et al., 2017). Although we focus only on the leader’s identity, not the follower’s, we adopt this approach because it allows us to argue that individuals co-create their respective leadership identities in relation to other signifiers (Petriglieri and Peshkam, 2022). Therefore, the relationship with followers, as well as that developed with other leaders, is seen here as fundamental to understanding how a leader defines themselves as such.

Understanding this process becomes especially relevant in contexts where leadership is increasingly characterized by different expectations and judgments from other signifiers (Zhang et al., 2015). As more people with whom they interact internalize leadership identities that are mutually recognized and collectively endorsed, leaders tend to develop more stable self-concepts regarding who they are in this relational role (Ashforth and Schinoff, 2016). By using a view of leadership identity as a product of an interactional process involving claiming and granting leader status, we highlight the role of the other in the process of constructing oneself as a leader.

In the specific case of diversity leaders in organizations, this perspective is especially relevant due to the variation in expectations that others have regarding who they are (Petriglieri and Peshkam, 2022). While some individuals seem to grant leadership and endorse the identity of leaders in this position, others contest it (Felix and Santana, 2024). This brings us to the concept of Identity Threat, a perceived discrepancy between an individual’s current identity and potential threats to that identity (Piening et al., 2020). These threats can originate from various sources, such as changes in the organizational environment, negative feedback, or encounters with information or events that challenge the individual’s self-image (Bataille and Vough, 2022). In the case of this article, they stem from a perception that others invalidate how the leader acts regarding diversity, for instance, by framing diversity differently or by not believing in the systemic need for inclusion (Primecz and Mahadevan, 2024). Faced with identity threats, individuals may manifest a variety of psychological and behavioral responses, which may include defense, denial, adaptation, or even redefinition of identity, all with the aim of restoring a sense of coherence and self-esteem (Brown, 2022). This reinforces the importance of understanding leaders’ identities in a given position and the types of identity threats they experience.

### 2.2 Marginality in relation to leadership

Marginality is a key concept in sociology and psychology. It was originally put forward as an analytical unit by cultural sociologist Robert Ezra Parks in his conceptualization of the “marginal man” and systematized by Parks’ student Everett V. Stonequist (Park, 1937). According to Park (1928), marginal men are those who participate in the traditions of two peoples and who are in the frequent process of crossing cultures and social identity groups, e.g., because of their having migrated from one country to another. Park developed this concept in the concept of migration and immigration to the United States to understand how ‘being American’ would look like from the margins. Later, this approach was further developed by managerial disciplines, for instance, to understand how leaders with hybrid (in-between) identities might be better leaders because of their dual belonging and dual access to interpretive schemes (e.g., Mahadevan, 2015). It was also made apparent that not all “leader-identity duality” is equally valued in a certain context and by a certain organization. For instance, those leaders who have migrated from non-Western countries to the West or are racialized as non-White are more likely to be perceived as a presumably unqualified “migrant” compared to a Western and white manager moving to the non-West who is more likely to be identified as a high-skilled expatriate (Zanoni et al., 2010). Likewise, women leaders, in particular if they are racialized as non-White, are more likely to be perceived as “bad leaders” even though they might be highly innovative and effective not despite but *because* of them ‘not fitting in’ (their marginality, see Mayer et al., 2018). Thus, marginal leaders are those whose identity-duality, and, thus: their competencies and skills, are undervalued or neglected by a certain majority group or organizational context. Example for marginal leaders are, for instance, individuals from the non-West acting as global leaders (Fitzsimmons et al., 2013), bicultural leaders whose biculturality is not valued by the organization they work for and those whom they interact with Mahadevan (2015) and generally those whose alternative identities are thought of as not being a relevant source of leadership skills and competencies, based on dominant ideas of what constitutes “good leadership” and a dominant image of the “ideal leader” (Guttormsen and Lauring, 2018). Likewise, in the domain of leadership, some functions and professions are often deemed to be more relevant, such as strategic management, than others, such as managing diversity, equality and inclusion (Primecz and Mahadevan, 2024). For example, diversity area leaders recurrently need to defend in the necessity of their leadership positions—and, thus: also themselves as being in the position of “leaders”—, for instance, by offering better career opportunities for individuals belonging to historically disadvantaged groups (Van Knippenberg et al., 2020).

When managing their marginality, marginal leaders who wish to make an impact, thus need to find way of meeting the expectations of a dominant group while at the same time maintaining their access to their unique resource and skills base that is: their marginality. Those who assimilate completely, will ultimately loose access to the benefits of integrating dual identities, as, for instance, research on successful bicultural leaders suggests (Fitzsimmons et al., 2013). Furthermore, silencing marginal leaders is assumed to result in organizational losses in skills and competencies (Guttormsen and Lauring, 2018). However, because organizations are often unaware of how they might profit from the competencies of marginal leaders, it becomes the marginal leaders’ responsibility to manage their own marginality the tension of maintaining uniqueness and integrating themselves. Thus, what they need to do is to find ways in how their identity is validated by those who are representatives of the majority or more favored identity in the context the seek to influence. For instance, diversity leaders often need to defend the necessity of their roles in the face of colleagues more oriented toward economic and financial performance (Park, 2020; Park and Liang, 2020; Primecz and Mahadevan, 2024).

### 2.3 Leadership and diversity ambiguity

Our research, characterized by its qualitative approach, initially focused on an executive population dedicated to diversity and inclusion activities that, despite their growing influence, had received little academic attention (Paluch and Shum, 2023; Ng et al., 2021). Throughout the process, we identified the interconnection between informants’ narratives about their struggles to adapt to the organizational environment and articulate a diversity philosophy, and academic work on leader identity and diversity paradoxes (McBride et al., 2024). We also noted that the study of leadership has evolved toward a more dynamic and contextualized understanding, moving away from unilateral and hierarchical views (Petriglieri and Peshkam, 2022). There is a growing perspective that leaders are seen as individuals who emerge and operate in social interactions, with their identity playing a crucial role (McBride et al., 2024). The attainment and maintenance of this identity require ongoing work to define and validate the leader role, influencing their actions and leadership opportunities (DeRue et al., 2009).

The literature also highlights the inherent ambiguity of diversity in organizations, which can vary depending on the context and be shaped by different perspectives (Nadiv and Kuna, 2020). The predominance of unilateral perspectives sometimes focused on humanistic values, sometimes on pragmatic values, has been criticized, while there is a growing need to recognize and explore the diverse facets of diversity in research and organizational practice (Post et al., 2021). Marginal leaders, such as those in Diversity and Inclusion roles in Organizations, face unique challenges in dealing with the ambiguity of their functions and identities. They do not fully fit into dominant norms and often face conflicts when defining their role and purpose within the organization (Petriglieri and Peshkam, 2022).

Reflection on diversity, especially among cultural and individual aspects, emerges as a significant focus area (Meng et al., 2023). While organizations seek to balance the assimilation of different cultures with the promotion of individuality, leaders responsible for promoting diversity face the challenge of dealing with inherent paradoxes of organizational diversity (Nadiv and Kuna, 2020). Although several facets of diversity reflections in organizations have been identified, there is a gap in understanding how leaders face and integrate these demands for humanistic values and performance in their practice (Konrad et al., 2021). Investigating how diversity executives in Organizations tackle these challenges can provide valuable insights into effectively addressing the complexities of diversity in contemporary organizations.

### 2.4 Boundary theory

Marginal leaders in the diversity field often need to choose how to deal with the ambiguities involved in the exercise of their role (Petriglieri and Peshkam, 2022). In the present study, we used Boundary Theory as a sensitizing approach to understand the dynamics between potential emerging leadership identities. Boundary Theory discusses how individuals categorize objects, spaces, subjects, and interactions to construct, maintain, and alter boundaries that delimit different domains in life (Ashforth et al., 2000). This theoretical perspective has been applied, for example, in studies on identity management in family businesses (Knapp et al., 2013) and professional and personal identities (Felix et al., 2018; Felix et al., 2023c; Araujo et al., 2015).

According to this theory, boundaries separate domains from each other, characterizing themselves as symbolic co-constructions that determine where different aspects of our existence begin and end, such as private and public life or work and home, for example (Provost Savard and Dagenais-Desmarais, 2023). A domain is a “cognitive space that includes what is included within the space delimited by a boundary” (Kreiner et al., 2006). For example, workdays and weekends or coworkers and personal friends can be seen as opposing domains that are separated from each other with distinct degrees of permeability—the degree to which aspects of one domain connect with aspects of another (Clark, 2000).

Thus, a leader responsible for the Diversity area may create impermeable boundaries—thick and strong—between humanistic or pragmatic/performance purposes, meaning that this person would tend to prioritize one purpose over the other, segmenting them. However, this person could also adopt permeable boundaries (thin, weak) between these two approaches, meaning that they would tend to integrate these two purposes (Petriglieri and Peshkam, 2022). However, in real life, boundaries manifest as opposite dimensions of a continuum, rather than as bipolar categories (Ashforth et al., 2000), indicating that individuals may adopt intermediate levels of preferences for integrating or segmenting domains.

## 3 Methods

In this research, we employed the grounded theory method (Glaser and Strauss, 1967), conducting a qualitative and inductive study with executives responsible for Diversity and Inclusion in 66 organizations in Brazil, focusing on their experience as leaders. Qualitative methodology involving active categorizations are generally accepted to be the method of choice for studying novel phenomena (Mahadevan et al., 2024). Consequently, we chose this method due to the limited prior theorizing on the phenomenon. Thus, the literature presented earlier does not serve as confirmatory theory but rather as a sensitizing approach that illuminated our fieldwork. Our theoretical and methodological choices are based on ontological constructs grounded in constructivism, wherein reality is considered subjective and socially constructed. In epistemological terms, the study is interpretive and focuses on understanding phenomena from the perspective of how individuals make meaning of their experiences.

### 3.1 Selection of interviewees, sample demographics, and data collection

We opted for a semi-structured strategy in conducting the interviews. Thus, the protocol we used did not represent a word-for-word script but rather a general guide with questions about aspects such as motivations and personal expectations in the role, organizational support to perform their activities, and perceptions of success or failure in the role (for more details, see Table 1). The interviews were recorded with permission and transcribed professionally.

**Table 1:** Interview protocol.

Could you talk a bit about your professional journey up to this point?
What motivated you to start working with Diversity?
How do you train and stay updated for this position?
Have your expectations related to working in this position been met? Please explain.
Explain what the hierarchical position of your position is in the organizational structure.
What is your current position? How long have you held this position? And how long have you been with this organization?
What are your responsibilities in this position?
How many people do you lead? What are their roles? What is the role of your immediate supervisor?
What are the main challenges of your position? How do you seek to overcome them? Do you get help from anyone in overcoming these challenges? Who are these people, and what kind of help is offered?
Is there organizational support for your position? If yes, what kind of support? Have there been changes over time to reach this current scenario?
Could you provide an example of a moment when you felt successful in the position?
Could you provide an example of a moment when you felt unsuccessful in the position?
What diversity initiatives/practices do you offer? Could you provide examples?
How does the company you work for seek to promote work and offer development opportunities for historically disadvantaged people? Do you believe these practices are successful in their purposes? Could you justify?
What would be a successful diversity policy/practice for you? Could you provide an example?
Is your philosophy regarding diversity in organizations shared by members of your organization? In what aspects yes, in what aspects no? Who supports and who does not support?
How do you expect diversity and inclusion policies/practices in organizations to evolve over time?

The choice to examine the case of leaders responsible for Diversity and Inclusion in organizations operating in Brazil is due to two main aspects. First, the existence in the country of a highly mixed and unequal population (Mesquita and Bezerra, 2021), which increases the relevance of the Diversity and Inclusion area. Second, the characterization of the Diversity and Inclusion area as an example of Marginal Leadership (Dennissen et al., 2019; Janssens and Steyaert, 2019; Petriglieri and Peshkam, 2022).

Interviewees were recruited from a list of 550 Diversity and Inclusion leaders obtained from a database of an event focused on the theme. When composing the list, participants indicated that they allowed potential contacts for participation in research. This list was provided by a nonprofit organization that trains leaders for such a role. Leaders were invited by email to participate in the research. In the email, we introduced the concept of Marginal Leadership and asked that only those who identified with it express their intention to collaborate in the study. Among the invitees, 43 responded positively, were interviewed, and indicated another 23 individuals through the snowball sampling method (von Borell de Araujo et al., 2014). Thus, although most of the interviewees are characterized by individuals seeking training through this organization, we conducted a relevant number of interviews with individuals from other sources, leading us to a wide range of perspectives.

Interviewees varied widely in terms of age, race, gender, experience in the role, and in the job market overall. The ages of the interviewees ranged from 31 to 66 years, with an average of 46.8 years. Time of experience in the role ranged from 2 (minimum criterion) to 8 years, with most interviewees being female (70.2%). Most interviewees (56.6%) are homosexual, mixed-race (47.5%), and have been active in the job market for 4–38 years. In line with the principles of theoretical sampling, we sought to identify interviewees with diverse characteristics, which allowed us to continually compare whether the patterns identified in some interviews were repeated in subsequent observations. Thus, the principle of theoretical sampling guided the field access strategy.

This study was conducted following the recommendations of the FBSR Guidelines, an ethics committee established at the Fucape Business School, with written informed consent from all subjects. All subjects gave written informed consent in accordance with the Declaration of Helsinki. The protocol was approved by an ethics committee established at the Fucape Business School.

### 3.2 Data analysis

**Figure 1:**
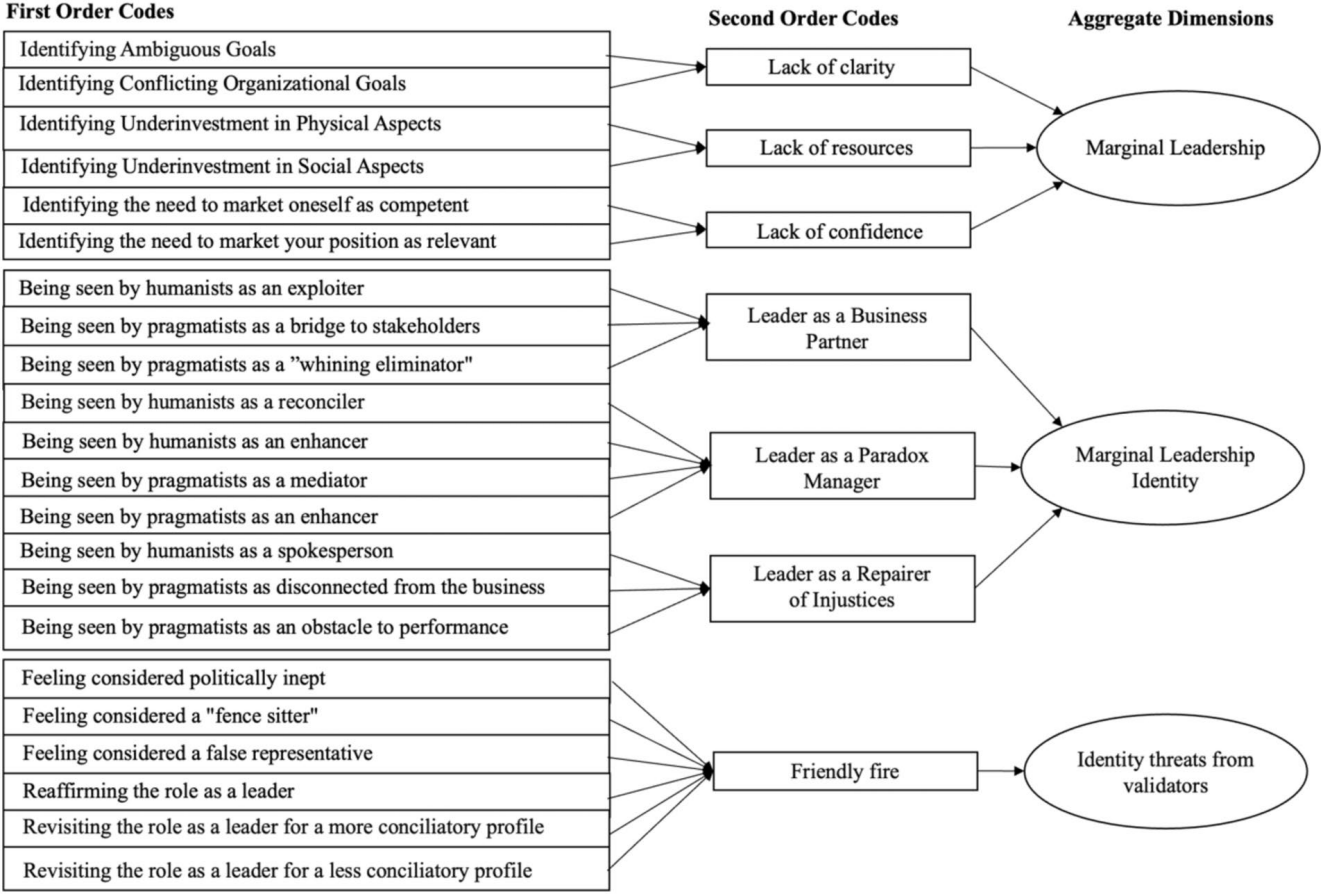
Data structure.

During the iterative process of data collection and analysis, we generated a set of memos and three levels of codes (e.g., Gioia et al., 2013). First-order codes typically began with gerund verbs and were used to describe what we interpreted as occurring in the data. These initial codes were grouped into second-order codes, which were more abstract and less descriptive in their meanings. Finally, the second-order codes were grouped into aggregated dimensions, which are codes of broader theoretical significance (Eisenhardt et al., 2016) and underpinned the construction of our theoretical model. This process concluded when further data collection and analysis were no longer able to generate relevant codes (theoretical saturation), and the most central categories were arranged into a theoretical model, as presented in the results section. The interview protocol ceased to be revised after interviewing 52 individuals, and no new codes emerged after interview number 56. The coding process was conducted independently by two authors of the article. In cases where there was disagreement between them on which code a specific segment of the interviews should be classified under, one of the other authors acted as an independent judge and provided interpretations that helped to reach a consensus. This process led us to significant theoretical insights and an analysis less dependent on the individual assumptions of the first two authors. The overall agreement rate between the two authors was 91%, above the desired standard of 70% suggested (Cohen, 1960). Figure 1 illustrates the data structure derived from this coding process, which formed the central categories underpinning the construction of our model.

## 4 Results

**Figure 2:**
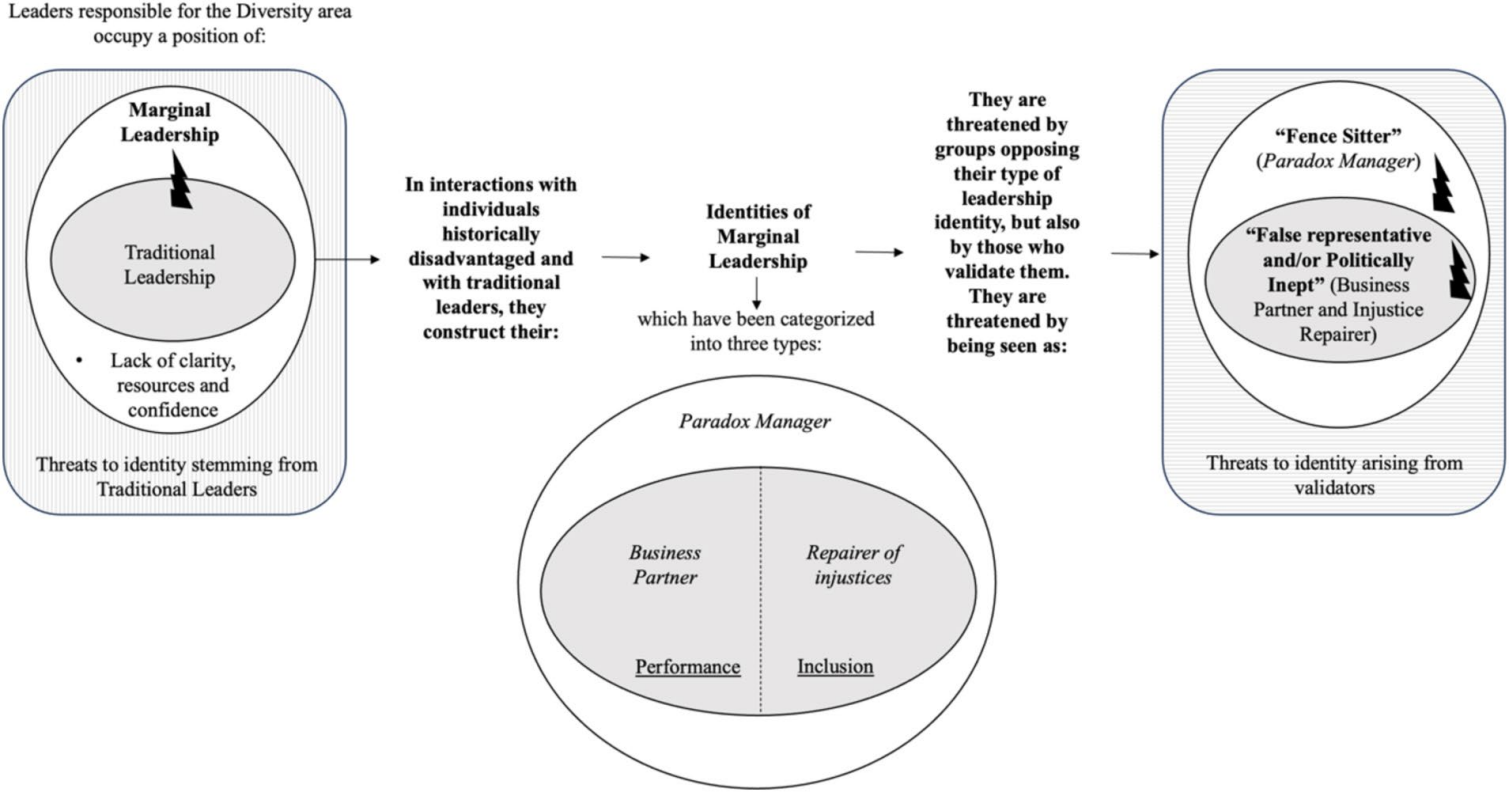
Theoretical model.

In this section, we present the substantive theory we constructed to understand the different forms of Marginal Leadership Identity experienced by executives responsible for Diversity and Inclusion, as well as the threats to such identities. In our model, we theorize that individuals in positions of Marginal Leadership, where there is little clarity of role, resources, and confidence to exercise their function, face threats to their leadership identity from Traditional Leaders. Additionally, we identified that, in interaction with individuals representing the historically disadvantaged groups to be promoted and Traditional Leaders, they can construct three types of Marginal Leadership: “Business Partner” (performance-focused), “Justice Repairer” (inclusion-focused), and “Paradox Manager” (performance and inclusion-focused). Finally, we also theorize that threats to their leadership identities vary according to the type of Marginal Leadership constructed. While “Business Partners” and “Justice Repairers” tend to be seen as false representatives or politically inept, the “Paradox Manager” tends to be labeled as a “Fence Sitter.” We detail each of these results below, synthesized in Figure 2.

### 4.1 Marginal leadership challenges and threats to identity from traditional leaders

Our initial findings concern the identity threats that the interviewed marginal leaders experience in their interactions with traditional leaders. Reports of a lack of clarity regarding their roles, resources to carry them out, and confidence to achieve their goals were recurrent.

The lack of clarity was mainly due to the fact that expectations regarding the role they play as leaders varied greatly depending on the leader they interacted with and the moment experienced by the organization. Several reports indicate that at times they were expected to promote diversity and inclusion as a value that exists not as a means, but as an end in itself. However, at other times, there was a widespread perception that they should promote diversity as long as it led to better impressions from clients, retention of key employees, or improvements in the organizational climate, for example. Thus, faced with this lack of clarity regarding their role as leaders in the diversity and inclusion area, the interviewees find themselves in situations where they see their selves as leaders threatened. The following report illustrates this finding:

“It’s a tremendous challenge to be a leader in the Diversity area. We’re not very sure what is expected. One moment they want us to promote well-being, they say that diversity is a value in itself. But just 1 month of poor results and they ask me how I’m going to make diversity profitable. Difficult. So, I always feel under scrutiny. You cannot simply focus on directing your action toward something because someone more powerful will criticize you at some point” (RP23).

A second factor that leads marginal leaders to feel threatened in their leadership identity is the lack of resources to carry out their work. Traditional leaders typically have sufficient people and financial resources, for example, to meet their goals of attracting clients, executing projects, and investing in production capacity. However, according to the interviewees, it was common to report that despite broad speeches of support for diversity, there was often a feeling that there were not enough resources to invest in diversity and inclusion, for example, when this requires greater investments in infrastructure change, technology development, and hiring more individuals in diversity positions. The following reports illustrate this:

“I’ve worked leading HR and now I’m dealing with Diversity issues. Talking with other leaders one day we discussed the budgets of our areas. Not only budgets, but also support availability, those things, to get things going. By far, I am the area with the least resources and that puts me in an inferior status, for sure” (RP47).

“It’s easy to say that you invest in diversity when it comes to making a poster for an event or when it comes to hiring a female manager. These specific things are easier. And when it comes to buying software so that blind employees can work? And when it comes to building ramps to improve the mobility of wheelchair users? At that moment, it becomes a big question mark, and then we see that insisting on these needs makes us stigmatized” (RP29).

In both cases, it is evident that leaders considered traditional often do not look favorably upon any insistence by leaders responsible for diversity and inclusion for greater investments in aspects such as infrastructure or equipment. According to several interviewees, this affects their self-esteem as leaders.

The third commonly found factor characterizing threats to the identity of marginal leaders refers to a lack of confidence. Leadership processes involve trust as they encompass assignments of power and responsibility to others. According to several interviewees, leaders in the more traditional areas of organizations, such as Finance, Production and Logistics, for example, are respected in their autonomy to carry out actions aligned with the organization’s purposes. However, reports were not uncommon that, as leaders responsible for diversity and inclusion in organizations, they do not feel they receive that same trust. Several interviewees said they noticed a lack of autonomy and a disbelief that they will act according to the organization’s purposes. For them, this positions them in a marginal status in the organization’s hierarchy and generates threats to identity originating from other leaders in more consolidated positions.

“I feel they do not trust me. They do not give autonomy, there’s a suspicion at every step I take. Everything needs to be reviewed. I feel they always fear that I say something that generates controversy, that I create a sense of protectionism, that I displease Greeks or Trojans. I am clearly a leader who is always in a lateral position, somewhat subordinate, subject to control” (RP50).

This report shows something recurrent in the data: the lack of trust involves both the risk of being overly protective of the causes of minority groups and of doing something that causes controversy or represents a gaffe or inability to understand the limits of how to act in favor of diversity.

Together, lack of clarity, lack of resources, and lack of confidence form a state of ambiguity that contrasts with the well-defined mandates of traditional leaders. Such aspects reinforces the peripheral status of our respondents, signaling a lack of commitment to their mission from the organization, which undermines their authority and agency. Thus, we grouped these threats as the aggregate dimension of marginal leadership.

### 4.2 Marginal leadership identities

Next, we sought to understand which identities are most commonly constructed among the interviewees as marginal leaders responsible for diversity and inclusion in organizations. Adopting a more relational perspective on leadership identity, we understand that they are constructed through a process whereby individuals make claims for a certain identity, which may or may not be validated by others. Similarly, followers also make claims for certain identities that may or may not be accepted.

In this context, we identified three types of identities most recurrent among the interviewees. The first refers to the leader as a “business partner,” who approaches the center of organizational power and aligns with the dominant identity of those who understand that the role of this area is to make the company profitable. In this case, the leader was seen by other managers and more pragmatic subordinates as bridges for better dialogue with stakeholders who have high demands for performance. Such pragmatism allowed them to build an identity associated with what we coded as “eliminator of complaints,” in other words, a diversity and inclusion leader who supposedly would not give voice to any demands from minorities considered excessive or protectionist, as exemplified below.

“I lead by results. I am a diversity leader, but I am here to keep the company alive and thriving. Because I think like this, I am seen differently from the last person who held this position (…). They see me as someone essential to end this victimhood, this bunch of protectionism and complaints that we know exists from minorities. It’s not that there is no prejudice and exclusion. We know there is. But what I fight against is the whining, and other leaders perceive that” (RP22).

On the other hand, these same individuals who adopt this pragmatic leadership identity were seen by those more sympathetic to the demands of historically disadvantaged groups as exploiters and defenders of the majority. This shows a certain heterogeneity in the constructed identities, revealing that the same identity tends to be valued and validated by one group while being stigmatized by others. The following reports illustrate this idea.

“I see myself as a diversity leader who needs to make people understand that diversity generates results. There is a diverse consumer market, and we have to have diversity in here to help us do better business with that market. It’s for everyone. But now and then, I see people saying that I’m an exploiter when I say that a person with a disability, a blind person, a woman, a gay person can be profitable. They say I’m trying to use people. They cannot think in terms of win/win” (RP8).

“In my management, my focus is to do something that is good for everyone. The company needs to profit, profit is good for everyone. This is a company, my role is to profit. And I try to do this through diversity, because of my position. It’s natural. Then I see that, because I act like this, several leaders from the more prominent areas see me as a partner, as someone they can count on to sell more, to produce better, to innovate. I am an ally to them” (RP56).

The second recurrent group concerning marginal leadership identities is “injustice repairers.” These individuals understand that in the historical colonization process of Brazil, a series of injustices were committed against blacks, indigenous peoples, women, and individuals belonging to other disadvantaged groups, such as people with physical disabilities and non-heterosexuals. Therefore, they advocate that their role in D&I leadership is essentially social and historical reparation-oriented, at the expense of profit as the main objective, as illustrated in the following quote.

“I make it clear, and everyone knows that I’m here to generate diversity, equity, and inclusion. To correct historical mistakes of our society, to the extent of my ability. That’s the person I am as a diversity leader. And it’s very gratifying for me to know that a lot of people here see me as a spokesperson. Just last week, a person who uses a wheelchair told me that and made my day count” (RP35).

According to the interviewees, profit may be a consequence of this reparation of a historical debt with the less privileged, but it does not represent the real purpose of their actions. Leaders in diversity and inclusion positions reported that they are seen as spokespeople by historically disadvantaged groups. On the other hand, they are also seen as disconnected from business or as an obstacle to performance. Thus, they lead from the margin, without identifying with traditional leaders. The following interview excerpt illustrates this categorization.

“I was hired for this position, and I know I am a leader here to really promote diversity, in the strongest sense of the word. To provide the conditions for people to feel included. That’s what I think, that’s how I am seen. And precisely because of this, I know that my CEO, the CFO, and the marketing director, who are the people who clash with me the most, see me as someone who is not concerned about profit, who is disconnected from their vibe, and who even hinders profit. They see me as an obstacle. They created the position just for show” (RP38).

“With me, sometimes it comes that my position was created just for show. There are some more empowered folks here who always invalidate me, who do not see me as someone who is helping. I came to work for diversity; it’s tough” (RP6).

The “injustice repairers,” therefore, understand that they are in the organization with the role of fixing historical mistakes in favor of equity and inclusion. For them, their leadership must occur independently, from a marginalized position, excluded from the spaces occupied by other leaders they call “traditional.”

Lastly, we categorized the third marginal leadership identity as “Paradox Manager.” We observed that such leaders do not make a clear choice between leading from the center of organizational power (“business partner”) or from the margin (“injustice repairers”). On the contrary, their identity reflects a belief in leading considering business partnership and injustice repair as a paradox to be managed. Thus, they understand that repairing injustices is a means to promote business and vice versa. The following interview excerpts show this.

“It’s not easy in this position because there are very varied expectations about who we have to be. I have a bit of a yin and yang thing, of thinking that I will not be just one thing or the other. I believe I need to help with profit, yes, but not by masking diversity actions. My role is to include so that people can be profitable. To profit so that we can include more disadvantaged people. That’s how I see myself (…). I think both those who support diversity, minorities, and people who are somewhat skeptical, let us say, see me as someone who empowers, who creates bridges both for the company to do well and for it, I do not know, to fulfill its social role, you know?” (RP63).

“For me, you have to associate and suck sugar cane. You have to create a fairer work environment, bring minorities in, but you also cannot just do that and think it’s okay. You have to make it happen. You have to show results. I emphasize organizational climate a lot, understanding how minorities think, so I think I’m a person who takes the corner kicks and heads, you know? (…) People who are more progressive tend to like me because they see me as someone who can create bridges, open doors, reconcile interests” (RP18).

These examples show expressions like “associate and suck sugar cane” (popular expression in Brazilian Portuguese) and “take corner kicks and head,” expressions from Brazilian popular language that represent the need to simultaneously perform activities that are usually seen as exclusive. As RP2 says, “you need a different mindset; I need to perform through diversity and I need to promote diversity with the resources I generate, I need to show results to expand and justify investments.” Thus, although both are leaders in diversity and inclusion, the way they constitute their identities in this position influences the place occupied by each leader in the performance of their function.

We understand “business partner,” “injustice repairer,” and “paradox manager” as attempts by our respondents to negotiate their hierarchical position and fulfill diversity and inclusion goals in ways that can be accepted—or resisted—by different organizational groups. These identities are developed as the interviewees engage in “claiming” and “granting” within their unique contexts, seeking validation from both traditional and minority-centered stakeholders (DeRue and Ashford, 2010). By navigating these divergent expectations, our respondents embody a leadership identity that simultaneously integrates and challenges the core values and goals of mainstream leadership. These three categories consequently form the aggregate dimension of “marginal leadership identity,” because they represent a distinct leadership identity construction process that occurs at the organizational periphery.

### 4.3 Threats to identity arising from validators of their marginal leadership identities

Despite common sense suggesting that the identity threats experienced by marginal leaders responsible for diversity and inclusion would be stigmatized only by groups opposing the polarity they defend, our results have shown a more complex reality. Threats to their identities were reported by individuals choosing to lead from the center of organizational power (business partners), those leading from the margin (injustice repairers), or those managing the paradox between performance and inclusion (paradox managers).

Surprisingly to us, threats to the identities experienced by “business partners” and “injustice repairers” proved to be similar. Such threats initially involve the accusation of “false representatives.” In these cases, social groups supporting their leadership roles also considered that they were not firm enough in defending a viewpoint underpinning the formation of that type of identity.

For example, in the following case, a leader who identifies as a business partner reports a perceived threat to his identity, as he is seen either as someone who does not sufficiently represent the interests of the supporting group of that identity, or who lacks the political ability to interpret the expected discourses when demanded:

“Look, I have a more proactive approach to diversity as a path to better performance, which is very true in our education sector. Marco [fictitious name] is an example of someone who thinks like me but who constantly challenges me. He says I should be more outspoken in this idea that the end goal is financial results. When I try to negotiate more, he says I am not representing the leadership’s interests well (…) But at the same time, he himself complained that I should have more political savvy, that I should have acted more politically when we performed poorly on a diversity indicator and the ESG team got upset. He thought that in this case, I had to find a way to approve more diversity actions. So the impression I have is that in this position, I will always receive criticism, either for being too soft and giving in to agendas or for not being good enough to approve diversity policies” (RP65).

In the context of these identity threats, complaints accumulate, whether because the employee is no longer “outspoken” in seeking to defend the view of diversity as a business partner or because they do not know how to position themselves politically in favor of this cause. It is curious to note that, in both situations, the criticisms are made by the individuals who would theoretically validate these identities. A similar situation occurs with leaders more grounded in the idea of “injustice repairers,” as shown in the following account.

“The most hurtful criticisms are from those I thought would support me (…). They are the people who benefit the most from my work, people from historically disadvantaged groups, but who end up finding fault in everything (…). Once, a black lesbian came to say that I, as a lesbian and diversity leader, only pretend to represent diversity because the beneficiaries of gender policies are usually white women, and the beneficiaries of racial policies are usually men and heterosexuals. She labeled me as a false representative. I understand her point, but I think things are gradually improving. On the other hand, there was also a woman who criticized me because she thinks I was too harsh in a case of harassment accusation against a woman, she thinks I lacked the ability to handle the situation (…) This hurts because criticism from those who benefit from how things are is already expected. But this was a surprise to me, at least the frequency with which it occurs” (RP12).

Similar to the previous example, the criticisms mainly stem from the perception that the leader in question either pretends to represent the issue of diversity or is too harsh in defending a woman in a possible harassment situation. Obviously, we do not intend to contest the criticisms or the leaders here, only to theorize about the interactions. In this sense, it seems to us that issues of intersectionality were part of the identity threat experienced.

Interestingly, the “Paradox Managers” also found themselves contested. In the following example, a leader who acts with this identity highlighted how people who fit into the minority position criticized her performance:

“There was a case like this: a guy who is gay said that I am a fence-sitter, that I should choose a side. That supporting gays and at the same time implying that I think sexual orientation diversity is good strategically for the company would be a contradiction (…) Certainly, this is very bad, it makes me feel kind of trapped” (RP61).

“The other day, a black woman came to me and said it was cool that I’m always talking about racial issues in hiring and career policies here at the company. And I smiled, thinking I was doing great. But then she said: ‘Now all you have to do is decide if you want to work for the poor or for the rich.’ I said I did not understand, and she said I had to get off the fence because while I was doing that, I also always say that diversity can be a path for the company to have better results (…) Yes, she sees me as someone who does not take a stand, sitting on the fence (…) I do not like that, of course, huh? It seems like my work is not recognized by those who should value it the most” (RP26).

Faced with these threats, followers could not see promotion of diversity and financial results as a paradox that can be managed, but rather as a dilemma. The identity threats reported in such situations reflect a view that a choice between both polarities is necessary. This vision is expressed mainly by the term “fence-sitter,” which has been used in the Brazilian political context to describe someone in the center.

It is important to emphasize that the constructed model was influenced by demographic variables, such as age, gender and sexual orientation. In particular, the concepts of marginal leadership, marginal leadership identity threats and identity of validators were influenced by such demographic issues. More specifically, in the case of women, older individuals and homosexuals, they were those who showed that they experienced the phenomena described more intensely, although this was not explored in greater detail for reasons of parsimony. Thus, future research can explore the role of demographic issues in the construction of the model.

## 5 Discussion

The objective of the present study was to understand how executives responsible for Diversity and Inclusion construct their identities while occupying positions of “Marginal Leadership,” and how they deal with threats to such identities. Next, we present the theoretical contributions and theoretical implications related to this study.

### 5.1 Theoretical contributions

This study has potential implications for the literature, notably in the field of role identities (Crocetti et al., 2014; Ma and Peng, 2019), considering that we used a boundary-based approach to explore how individuals help create a role identity in positions where there are no clear expectations. By adopting perspectives from Identity Theory (Stryker, 1980; Stryker and Burke, 2000) and Boundary Theory (Ashforth et al., 2000; Hall and Richter, 1988), we contribute to the literature on role identity by showing how the role identity of leaders responsible for diversity in organizations is influenced by individuals’ preferences for more integrative or segregating boundaries between the humanistic and pragmatic values that pressure their performance. Thus, we expand the discussion from “fitting into a role with pre-existing roles” (Ma and Peng, 2019) to “building a role from agentic behaviors.” This is fundamental in a scenario where more and more leaders will work in newly created positions to meet market transformation demands (Warrick and Cady, 2023; Galanti et al., 2023).

The study also contributes to the literature on leadership identity construction (Haslam et al., 2022). Generally, this literature tends to focus on the identity of professionals for whom there are clear expectations in terms of values (Smith et al., 2018). Thus, most of the literature on leadership identity tends to explore identity work processes, through which individuals manage the tension between expressing their self authentically and fitting social expectations (Felix et al., 2023a). We contribute to the literature by showing that, in the case of marginal leaders, the lack of a clearly established behavioral model demands that these professionals face the duality between humanism and pragmatism more consciously and authoritatively. We also show how these professionals engage in identity play processes (Ibarra and Petriglieri, 2010), through which they seek to experiment with provisional selves until they feel more secure in performing identities in situations where their positions are seen as riskier. Thus, by expanding the view on leadership identity construction to identity play, and not just identity work, we also contribute to a more process-oriented view on the subject.

Finally, the study also presents contributions to the literature on identity threats. Several studies have been conducted to analyze how individuals respond to threats to organizational identities (Piening et al., 2020) and occupational identities (Murphy and Kreiner, 2020; Felix et al., 2023a). On the other hand, some have also been dedicated to studying threats to individual identities in the work context (Felix et al., 2023b). In this study, we specifically insert ourselves into an existing debate about leadership identity (Felix and Santana, 2024; Lee Cunningham et al., 2023). We add to this literature by directing attention not to traditional and central positions in the organizational power structure, but to emerging and peripheral positions. Furthermore, we explore not only identity threats in which the source of the threat is prototypically oppositional groups, but also those coming from groups that would theoretically validate the identity in question. Thus, we offer a more realistic and non-stereotypical view of identity threats in the daily lives of workers.

Furthermore, we contribute to the field of Diversity & Inclusion in theory and practice. As a concept, diversity management originates from North America, especially the United States. It was suggested as a response to social tensions, especially racial and gender inequalities (Litvin, 1997). The aim was to achieve higher social equality and to work toward more equity, that is: equal opportunities of being included for more groups, by means of affirmative action. This brought about the question: how can organizations achieve this goal, and Lorbiecki (2001) proposed a learning perspective for doing so. Our study shows a concrete way in which this organizational learning can be achieved, namely by promoting those D&I marginal leader identities that are the most conducive toward achieving diversity change. As Mahadevan (2012) has shown in her empirical study of a closed struggling with diversity change, also identity-based resistance can be a means of achieving Diversity and Inclusion, if it leads to leaders rethinking and readjusting their identities in relation to what needs to be achieved. Consequently, assessing leader identity in relation to D&I goals requires qualitative methodologies, because, sometimes, the objective facts of the situation are less telling than how individuals construct themselves in light of the D&I challenges they face. Thus, the D&I challenge at work involves much more than diversity being a ‘must-have concept’ for organizations to remain competitive (Ely and Thomas, 2020): rather, it requires a profound renegotiation of identities and their boundaries, and our study has exemplified this process for the relevant D&I context of Brazil.

### 5.2 Implications for practice

This study has potential implications for practice. We believe that a better understanding of the concept of Marginal Leadership, proposed by Petriglieri and Peshkam (2022), and its diffusion, favors executives occupying such positions to have greater clarity about the tensions related to the performance of their roles. Due to unawareness, marginal leaders tend to ignore the fact that they are in a position less endowed with clarity, resources, and confidence, facing frustrations and challenges that could be anticipated.

The results of our study provide these leaders with a clearer conceptual map of how to act in the face of different identities and threats, in two main ways. First, leading from the center, either supporting a more pragmatic or humanistic polarity. Second, leading from the margin, with a paradoxical vision. Considering the pressing intentions under marginal leaders, being aware of the map of possible positions may allow such professionals to be better prepared to act in the face of conflicting demands.

Third, the model presented may also be useful for significant others who interact with marginal leaders—especially those who represent sources of validation for their marginal leadership identities—to rethink the level of demand and identity threats they may sometimes express. Threats from groups that are theoretically supportive often sound strongly discouraging to leaders and sources of discouragement and hopelessness that their work will make a positive difference. Thus, the findings of our study allow these individuals to assess their interactions and reflect on whether their observations are indeed pertinent or if they exert disproportionate and even unfair pressure on marginal leaders.

We also enable organizations to navigate the conflicts associated with the business case for diversity and the humanistic goals underlying D&I (see Primecz and Mahadevan, 2024), namely by focusing on the identity-processes required for achieving positive diversity change. We propose a concrete way in which the learning perspective on D&I (Lorbiecki, 2001) can be implemented in practice.

Finally, we re-position Marginal Leadership as an asset, not as a liability for organizations. It is not *despite* the conflicts associated with their identities that marginal D&I leaders succeed in working toward positive diversity change, but rather *because of it.* Thus, out study also underscores the relevance of the D&I goal of working toward higher equity and equality for historically and systemically disadvantaged groups: Their experiences of being perceived as ‘different’, ‘other’ or ‘not belonging’, and their consecutive need to engage in identity work, can now be redefined as the ‘engine’ through which positive diversity change may be achieved.

## 6 Final considerations

### 6.1 Conclusion

In our model, we theorize that leaders who occupy positions of Marginal Leadership, where there is little role clarity, resources, and confidence for the exercise of their function, face identity threats to their leadership identity that are not faced by Leaders in more traditional positions, such as Finance, Production, and Marketing. In addition, we identified that, in interaction with individuals who are categorized as representing those historically disadvantaged groups in need of promotion and with traditional leaders, they can build three types of Marginal Leadership: “Business Partner” (focus on performance—boundary segmentation), “Injustice Repairer” (focus on inclusion—boundary segmentation), and “Paradox Manager” (focus on performance and inclusion—boundary integration). Finally, we also theorize that threats to their leadership identities vary according to the type of Marginal Leadership constructed. While “Business Partners” and “Injustice Repairers” tend to be seen as false representatives or politically inept, the “Paradox Manager” tends to be labeled as a “Fence-sitter.” Thus, we provide a detailed view of the challenges to the identity built by Marginal Leaders responsible for diversity and inclusion in organizations.

### 6.2 Limitations and implications for future research

Despite this study’s potential contributions to theory and practice, some limitations, which may represent opportunities for future research, are worth noting.

We focused our interviews on Marginal Leaders responsible for Diversity and Inclusion in companies. However, diversity threats to marginal leaders may occur more broadly, encompassing individuals in other organizational functions. Thus, we suggest that research maps threats to the identity of Marginal Leadership experienced by other leaders responsible for areas such as Learning & Development, Workplace Safety and Environment, Social, and Government (ESG) (Petriglieri and Peshkam, 2022).

Because we focused on building rapport during interview conversations, we did not collect some specific information about the context of activities and job-related characteristics of the respondents. This limitation opens the possibility for future studies explore how aspects like tenure with the position, sector of activity, and span of control relate to how marginal leaders craft identities that gave meaning and direction to their work.

Our research occurred at a specific moment in time and space, namely, between 2022 and 2024. It was also applied in the context of Brazil, a highly mixed-race country that is considered, for example, one of the most attractive tourist destinations for people of different races and sexual orientations (von Borell de Araujo et al., 2014). It would be interesting to replicate this study in other contexts, with more homogeneous populations and greater restrictions on the idea of diversity and inclusion. Moreover, it is possible that the Diversity and Inclusion area will gain more political strength and become more strategic for organizations, thus no longer being so marginalized. Therefore, we suggest longitudinal research that maps the evolution of the meanings of this area vis-à-vis more traditional ones over time.

Finally, our study was limited to mapping diversity threats experienced by these individuals but did not study the responses individuals offer to these threats. The literature on coping responses to identity threats is extensive and can be very useful in understanding the consequences of such threats to the self. Especially, it would be interesting to analyze these responses over time, and beyond the traditional responses of either maintaining the threatened identity or resignifying it (Petriglieri, 2011). Recent studies have shown less dichotomous ways of responding to these threats (Felix et al., 2023b; Felix and Santana, 2024), which seems very promising, especially for the group of “Paradox Managers.”

## Data Availability

The raw data supporting the conclusions of this article will be made available by the authors, without undue reservation.

## References

[ref1] Araujo B. F. V. B. Tureta C. A. de Araujo D. A. V. B. (2015). How do working mothers negotiate the work-home interface? J. Manag. Psychol. 30, 565–581. doi: 10.1108/JMP-11-2013-0375

[ref2] Ashforth B. E. Kreiner G. E. Fugate M. (2000). All in a day's work: boundaries and micro role transitions. Acad. Manag. Rev. 25, 472–491. doi: 10.2307/259305

[ref3] Ashforth B. E. Schinoff B. S. (2016). Identity under construction: how individuals come to define themselves in organizations. Annu. Rev. Organ. Psych. Organ. Behav. 3, 111–137. doi: 10.1146/annurev-orgpsych-041015-062322

[ref4] Bataille C. D. Vough H. C. (2022). More than the sum of my parts: an intrapersonal network approach to identity work in response to identity opportunities and threats. Acad. Manag. Rev. 47, 93–115. doi: 10.5465/amr.2018.0026

[ref5] Brewer M. B. Gardner W. (1996). Who is this" we"? Levels of collective identity and self representations. J. Pers. Soc. Psychol. 71, 83–93. doi: 10.1037/0022-3514.71.1.83

[ref6] Brown A. D. (2022). Identities in and around organizations: towards an identity work perspective. Hum. Relat. 75, 1205–1237. doi: 10.1177/0018726721993910

[ref7] Carrillo Arciniega L. (2021). Selling diversity to white men: how disentangling economics from morality is a racial and gendered performance. Organization 28, 228–246. doi: 10.1177/1350508420930341

[ref9001] Clark S. C. (2000). Work/family border theory: A new theory of work/family balance. Human relations, 53, 747–770. doi: 10.1177/0018726700536001

[ref8] Cohen J. (1960). A coefficient of agreement for nominal scales. Educ. Psychol. Meas. 20, 37–46. doi: 10.1177/001316446002000104

[ref9] Collinson D. (2005). Dialectics of leadership. Hum. Relat. 58, 1419–1442. doi: 10.1177/0018726705060902

[ref10] Crocetti E. Avanzi L. Hawk S. T. Fraccaroli F. Meeus W. (2014). Personal and social facets of job identity: a person-centered approach. J. Bus. Psychol. 29, 281–300. doi: 10.1007/s10869-013-9313-x

[ref11] Dennissen M. Benschop Y. Van den Brink M. (2019). Diversity networks: networking for equality? Br. J. Manag. 30, 966–980. doi: 10.1111/1467-8551.12321

[ref12] DeRue D. S. Ashford S. J. (2010). Who will lead and who will follow? A social process of leadership identity construction in organizations. Acad. Manag. Rev. 35, 627–647. doi: 10.5465/AMR.2010.53503267

[ref13] DeRue D. S. Ashford S. J. Cotton N. C. (2009). “Assuming the mantle: unpacking the process by which individuals internalize a leader identity” in Exploring Positive Identities and Organizations Laura M.R and Jane E.D (Eds), (New York, NY, USA: Psychology Press), 217–236.

[ref14] Eisenhardt K. M. Graebner M. E. Sonenshein S. (2016). Grand challenges and inductive methods: rigor without rigor mortis. Acad. Manag. J. 59, 1113–1123. doi: 10.5465/amj.2016.4004

[ref15] Ely R. J. Thomas D. A. (2020). Getting serious about diversity. Harv. Bus. Rev. 98, 114–122.

[ref16] Epitropaki O. Kark R. Mainemelis C. Lord R. G. (2017). Leadership and followership identity processes: a multilevel review. Leadersh. Q. 28, 104–129. doi: 10.1016/j.leaqua.2016.10.003

[ref17] Felix B. Galon S. Z. Amaro R. D. A. (2023c). How do women balance multiple roles during the post-maternity-leave period? Community Work Fam. 1-18, 1–18. doi: 10.1080/13668803.2023.2199132

[ref18] Felix B. Júlio A. C. Rigel A. (2023a). ‘Being accepted there makes me rely less on acceptance here’: cross-context identity enactment and coping with gender identity threats at work for non-binary individuals. Int. J. Hum. Resour. Manag. 35, 1851–1882. doi: 10.1080/09585192.2023.2254211

[ref19] Felix B. Mello A. von Borell D. (2018). Voices unspoken? Understanding how gay employees co-construct a climate of voice/silence in organisations. Int. J. Hum. Resour. Manag. 29, 805–828. doi: 10.1080/09585192.2016.1255987

[ref20] Felix B. Santana J. (2024). Under pressure: how leaders react to identity threats related to their paradoxical leadership. Cadernos EBAPE. BR 21:e2022. doi: 10.1590/1679-395120220154

[ref21] Felix B. Tiussi B. L. Mahadevan J. Dias R. C. (2023b). The great pretenders? Individuals’ responses to threats to their remote worker identities. Front. Psychol. 14:1224548. doi: 10.3389/fpsyg.2023.1224548, PMID: 38022977 PMC10657870

[ref22] Fitzsimmons S. R. Lee Y.-T. Brannen M.-Y. (2013). Demystifying the myth about marginals: Implications for global leadership. Eur. J. Int Magt, 7, 587–603. doi: 10.1504/EJIM.2013.056479

[ref23] Galanti T. De Vincenzi C. Buonomo I. Benevene P. (2023). Digital transformation: inevitable change or sizable opportunity? The strategic role of HR Management in Industry 4.0. Administr. Sci. 13:30. doi: 10.3390/admsci13020030

[ref24] Gioia D. A. Corley K. G. Hamilton A. L. (2013). Seeking qualitative rigor in inductive research: notes on the Gioia methodology. Organ. Res. Methods 16, 15–31. doi: 10.1177/1094428112452151

[ref25] Glaser B. G. Strauss A. L. (1967). The discovery of grounded theory: Strategies for qualitative research.

[ref26] Greene A. M. Kirton G. (2023). “Doing the right thing” and “making a difference”: the role of personal ethical values in diversity and inclusion consulting. J. Bus. Ethics 193, 179–191. doi: 10.1007/s10551-023-05514-w

[ref27] Guttormsen D. S. A. Lauring J. (2018). Fringe voices in cross-cultural management research: silenced and neglected? Int. Stud. Manag. Organ. 48, 239–246. doi: 10.1080/00208825.2018.1480465

[ref28] Hall D. T. Richter J. (1988). Balancing work life and home life: what can organizations do to help? Acad. Manag. Perspect. 2, 213–223. doi: 10.5465/ame.1988.4277258

[ref29] Haslam S. A. Gaffney A. M. Hogg M. A. Rast D. E. III Steffens N. K. (2022). Reconciling identity leadership and leader identity: a dual-identity framework. Leadersh. Q. 33:101620. doi: 10.1016/j.leaqua.2022.101620

[ref30] Ibarra H. Petriglieri J. L. (2010). Identity work and play. J. Organ. Chang. Manag. 23, 10–25. doi: 10.1108/09534811011017180

[ref31] Janssens M. Steyaert C. (2019). A practice-based theory of diversity: Respecifying (in) equality in organizations. Acad. Manag. Rev. 44, 518–537. doi: 10.5465/amr.2017.0062

[ref32] Knapp J. R. Smith B. R. Kreiner G. E. Sundaramurthy C. Barton S. L. (2013). Managing boundaries through identity work: the role of individual and organizational identity tactics. Fam. Bus. Rev. 26, 333–355. doi: 10.1177/0894486512474036

[ref33] Konrad A. M. Richard O. C. Yang Y. (2021). Both diversity and meritocracy: managing the diversity-meritocracy paradox with organizational ambidexterity. J. Manag. Stud. 58, 2180–2206. doi: 10.1111/joms.12752

[ref34] Kreiner G. E. Hollensbe E. C. Sheep M. L. (2006). On the edge of identity: boundary dynamics at the interface of individual and organizational identities. Hum. Relat. 59, 1315–1341. doi: 10.1177/0018726706071525

[ref35] Lee Cunningham J. Sonday L. Ashford S. J. (2023). Do I dare? The psychodynamics of anticipated image risk, leader-identity endorsement, and leader emergence. Acad. Manag. J. 66, 374–401. doi: 10.5465/amj.2018.1258

[ref36] Litvin D. R. (1997). The discourse of diversity: from biology to management. Organization 4, 187–209. doi: 10.1177/135050849742003

[ref37] Lorbiecki A. (2001). Changing views on diversity management: the rise of the learning perspective and the need to recognize social and political contradictions. Manag. Learn. 32, 345–361. doi: 10.1177/1350507601323004

[ref39] Mahadevan J. (2012). Utilizing identity-based resistance for diversity change: a narrative approach. J. Organ. Chang. Manag. 25, 819–834. doi: 10.1108/09534811211280582

[ref40] Mahadevan J. (2015). Understanding the process of intercultural negotiations through liminality: insights on bi-culturality, marginality and cultural expertise from a Sino-German business context. Int. J. Cross-cult. Manag. 15, 239–258. doi: 10.1177/1470595815601877

[ref41] Mahadevan J. Reichert T. Steinmann J. Stärkle A. Metzler S. Bacher L.. (2024). COVID-induced virtual teams: a phenomenon-based framework and methodological advice for studying novel events. Centr. Eur. Manag. J. 32, 262–283. doi: 10.1108/CEMJ-12-2022-0244

[ref38] Ma J. Peng Y. (2019). The performance costs of illegitimate tasks: the role of job identity and flexible role orientation. J. Vocat. Behav. 110, 144–154. doi: 10.1016/j.jvb.2018.11.012

[ref43] Martins L. L. (2020). Strategic diversity leadership: the role of senior leaders in delivering the diversity dividend. J. Manag. 46, 1191–1204. doi: 10.1177/0149206320939641

[ref44] Mayer C.-H. Surtee S. Mahadevan J. (2018). South African women leaders, transformation and diversity conflict intersections. J. Organ. Chang. Manag. 31, 877–894. doi: 10.1108/JOCM-10-2016-0196

[ref45] McBride A. Howe L. C. Gooty J. Banks G. C. (2024). Seeing with counterfactual lenses: alternative assumptions at the intersection of leadership and identity. Leadersh. Q. 35:101769. doi: 10.1016/j.leaqua.2023.101769

[ref46] Meng W. Xu Z. Abuliezi Z. Lyu Y. Zhang Q. (2023). Paradoxical leadership, team adaptation and team performance: the mediating role of inclusive climate. Front. Psychol. 14:1052732. doi: 10.3389/fpsyg.2023.1052732, PMID: 37089731 PMC10117128

[ref47] Mesquita J. S. Bezerra M. S. (2021). “Brazil cannot stop”: meritocratic ideology in an unequal country. Gend. Work. Organ. 28, 446–460. doi: 10.1111/gwao.12589

[ref49] Murphy C. Kreiner G. E. (2020). Occupational boundary play: crafting a sense of identity legitimacy in an emerging occupation. J. Organ. Behav. 41, 871–894. doi: 10.1002/job.2473

[ref50] Nadiv R. Kuna S. (2020). Diversity management as navigation through organizational paradoxes. Equality Divers. Inclus. Int. J. 39, 355–377. doi: 10.1108/EDI-12-2018-0236

[ref51] Ng E. S. Sears G. J. Arnold K. A. (2021). Exploring the influence of CEO and chief diversity officers' relational demography on organizational diversity management: an identity-based perspective. Manag. Decis. 59, 2583–2605. doi: 10.1108/MD-01-2019-0135

[ref52] Nkomo S. M. Bell M. P. Roberts L. M. Joshi A. Thatcher S. M. (2019). Diversity at a critical juncture: new theories for a complex phenomenon. Acad. Manag. Rev. 44, 498–517. doi: 10.5465/amr.2019.0103

[ref53] Noon M. Ogbonna E. (2021). Controlling management to deliver diversity and inclusion: prospects and limits. Hum. Resour. Manag. J. 31, 619–638. doi: 10.1111/1748-8583.12332

[ref54] Paluch R. M. Shum V. (2023). The non-white standard: racial bias in perceptions of diversity, equity, and inclusion leaders. J. Appl. Psychol. 109, 971–986. doi: 10.1037/apl0001106, PMID: 37289529

[ref55] Park R. E. (1928). Human migration and the marginal man. Am. J. Sociol. 33, 881–893. doi: 10.1086/214592

[ref56] Park R. E. (1937) in “Cultural Conflict and the Marginal Man”, Introduction to “The Marginal Man”. ed. Stonequist E. V. (New York, NY, USA: Charles Scribner's Sons), 372–376.

[ref57] Park S. (2020). Size matters: toward a contingency theory of diversity effects on performance. Public Perform. Manag. Rev. 43, 278–303. doi: 10.1080/15309576.2019.1657917

[ref58] Park S. Liang J. (2020). Merit, diversity, and performance: does diversity management moderate the effect of merit principles on governmental performance? Public Personnel Manag. 49, 83–110. doi: 10.1177/0091026019848459

[ref60] Petriglieri G. Peshkam A. (2022). Stranger leaders: a theory of marginal leaders’ conception of learning in organizations. Acad. Manag. J. 65, 1240–1273. doi: 10.5465/amj.2019.0162

[ref59] Petriglieri J. L. (2011). Under threat: responses to and the consequences of threats to individuals' identities. Acad. Manag. Rev. 36, 641–662. doi: 10.5465/AMR.2011.65554645

[ref61] Piening E. P. Salge T. O. Antons D. Kreiner G. E. (2020). Standing together or falling apart? Understanding employees’ responses to organizational identity threats. Acad. Manag. Rev. 45, 325–351. doi: 10.5465/amr.2016.0457

[ref62] Post C. Muzio D. Sarala R. Wei L. Faems D. (2021). Theorizing diversity in management studies: new perspectives and future directions. J. Manag. Stud. 58, 2003–2023. doi: 10.1111/joms.12779

[ref63] Primecz H. Mahadevan J. (2024). Intersectionality as a conceptual lens for advancing diversity, equity and inclusion in international business studies: newer developments from critical cross-cultural management studies and their insights for the business case. Crit. Perspect. Int. Bus. doi: 10.1108/cpoib-04-2022-0034

[ref64] Provost Savard Y. Dagenais-Desmarais V. (2023). Work-family spillover of satisfaction: the moderating role of domain boundary strength and identity salience. J. Occup. Organ. Psychol. 96, 599–623. doi: 10.1111/joop.12434

[ref66] Sluss D. M. Ashforth B. E. (2007). Relational identity and identification: defining ourselves through work relationships. Acad. Manag. Rev. 32, 9–32. doi: 10.5465/amr.2007.23463672

[ref67] Smith P. Haslam S. A. Nielsen J. F. (2018). In search of identity leadership: an ethnographic study of emergent influence in an interorganizational R&D team. Organ. Stud. 39, 1425–1447. doi: 10.1177/0170840617727781

[ref68] Spaaij R. Knoppers A. Jeanes R. (2020). “We want more diversity but…”: resisting diversity in recreational sports clubs. Sport Manag. Rev. 23, 363–373. doi: 10.1016/j.smr.2019.05.007

[ref69] Stryker S. (1980). Symbolic Interactionism: A Social Structural Version. Menlo Park, Menlo Park, CA, USA: Benjamin/Cummings.

[ref70] Stryker S. Burke P. J. (2000). The past, present, and future of an identity theory. Soc. Psychol. Q. 63, 284–297. doi: 10.2307/2695840

[ref71] Uhl-Bien M. Riggio R. E. Lowe K. B. Carsten M. K. (2014). Followership theory: a review and research agenda. Leadersh. Q. 25, 83–104. doi: 10.1016/j.leaqua.2013.11.007

[ref72] Van Knippenberg D. Nishii L. H. Dwertmann D. J. (2020). Synergy from diversity: managing team diversity to enhance performance. Behav. Sci. Policy 6, 75–92. doi: 10.1177/237946152000600108

[ref73] von Borell de Araujo B. F. Teixeira M. L. M. da Cruz P. B. Malini E. (2014). Understanding the adaptation of organisational and self-initiated expatriates in the context of Brazilian culture. Int. J. Hum. Resour. Manag. 25, 2489–2509. doi: 10.1080/09585192.2012.743470

[ref74] Warrick D. D. Cady S. H. (2023). Is your organization prepared to manage tsunami change? J. Appl. Behav. Sci. 59, 337–340. doi: 10.1177/00218863221132314

[ref9002] Zanoni P. Janssens M. Benschop Y. Nkomo S. (2010). Guest editorial: Unpacking diversity, grasping inequality: Rethinking difference through critical perspectives. Organization, 17, 9–29. doi: 10.1177/1350508409350344

[ref75] Zhang Y. Waldman D. A. Han Y. L. Li X. B. (2015). Paradoxical leader behaviors in people management: antecedents and consequences. Acad. Manag. J. 58, 538–566. doi: 10.5465/amj.2012.0995

